# New insights into the evolution of subtilisin-like serine protease genes in Pezizomycotina

**DOI:** 10.1186/1471-2148-10-68

**Published:** 2010-03-09

**Authors:** Juan Li, Li Yu, Jinkui Yang, Linqian Dong, Baoyu Tian, Zefen Yu, Lianming Liang, Ying Zhang, Xu Wang, Keqin Zhang

**Affiliations:** 1 Laboratory for Conservation and Utilization of Bio-resources, and Key Laboratory for Microbial Resources of the Ministry of Education, Yunnan University, Kunming, 650091, PR China; 2 Institute of Environment Sciences and Lakes Research, Yunnan University, Kunming, 650091, PR China; 3 Engineering Research Center of Industrial Microbiology of Ministry of Education, Fujian Normal University, Fuzhou, 350108, PR China; 4 School of Life Sciences, Yunnan Normal University, Kunming, 650092, PR China

## Abstract

**Background:**

Subtilisin-like serine proteases play an important role in pathogenic fungi during the penetration and colonization of their hosts. In this study, we perform an evolutionary analysis of the subtilisin-like serine protease genes of subphylum Pezizomycotina to find if there are similar pathogenic mechanisms among the pathogenic fungi with different life styles, which utilize subtilisin-like serine proteases as virulence factors. Within Pezizomycotina, nematode-trapping fungi are unique because they capture soil nematodes using specialized trapping devices. Increasing evidence suggests subtilisin-like serine proteases from nematode-trapping fungi are involved in the penetration and digestion of nematode cuticles. Here we also conduct positive selection analysis on the subtilisin-like serine protease genes from nematode-trapping fungi.

**Results:**

Phylogenetic analysis of 189 subtilisin-like serine protease genes from Pezizomycotina suggests five strongly-supported monophyletic clades. The subtilisin-like serine protease genes previously identified or presumed as endocellular proteases were clustered into one clade and diverged the earliest in the phylogeny. In addition, the cuticle-degrading protease genes from entomopathogenic and nematode-parasitic fungi were clustered together, indicating that they might have overlapping pathogenic mechanisms against insects and nematodes. Our experimental bioassays supported this conclusion. Interestingly, although they both function as cuticle-degrading proteases, the subtilisin-like serine protease genes from nematode-trapping fungi and nematode-parasitic fungi were not grouped together in the phylogenetic tree. Our evolutionary analysis revealed evidence for positive selection on the subtilisin-like serine protease genes of the nematode-trapping fungi.

**Conclusions:**

Our study provides new insights into the evolution of subtilisin-like serine protease genes in Pezizomycotina. Pezizomycotina subtilisins most likely evolved from endocellular to extracellular proteases. The entomopathogenic and nematode-parasitic fungi likely share similar properties in parasitism. In addition, our data provided better understanding about the duplications and subsequent functional divergence of subtilisin-like serine protease genes in Pezizomycotina. The evidence of positive selection detected in the subtilisin-like serine protease genes of nematode-trapping fungi in the present study suggests that the subtilisin-like serine proteases may have played important roles during the evolution of pathogenicity of nematode-trapping fungi against nematodes.

## Background

Subtilisin-like serine proteases play an important role in the pathogenicity of pathogenic fungi. By using subtilisin-like serine proteases, pathogenic fungi disrupt the physiological integrity of the hosts during penetration and colonization [1,2]. Previous studies have suggested that pathogenic fungi with different life styles utilize subtilisin-like serine proteases as their virulence factor [2-5]. Investigations into the similarities and differences of pathogenic mechanisms among these pathogenic fungi will significantly enhance our understanding of the evolution of these genes.

Pezizomycotina, the largest subphylum of Ascomycota, includes all filamentous, sporocarp-producing species. These organisms have diverse ecological niches and life styles [6]. A recent study based on six nuclear genes divided Pezizomycotina into nine major clades [7]. Among them, nematode-trapping fungi, which are conidial anamorphs of Orbiliomycetes and belong to nematophagous fungi, are unique because they capture free-living soil nematodes using trapping devices (e.g. adhesive networks, constricting rings, adhesive columns, adhesive knobs and nonconstricting rings) [8,9]. In contrast, nematode-parasitic fungi, another group of nematophagous fungi within Pezizomycotina, infect nematodes mainly using extracellular enzymes (subtilisin-like serine protease, chitinase and etc.) [2,10]. Previous studies have suggested that subtilisin-like serine proteases are involved in the penetration and the digestion of nematode cuticles [11-23]. However, only limited studies have been done on the evolutionary pattern of subtilisin-like serine protease genes in nematode-trapping fungi so far [10,11,17]. In view of this, we here newly determined five subtilisin-like serine protease genes from the nematode-trapping fungi. In conjunction with the other Pezizomycotina sequences obtained from previous studies and extensive database searching of available genome assembly, we perform the most comprehensive investigation to date of subtilisin-like serine protease genes in Pezizomycotina.

## Results

### New subtilisin-like serine protease genes from nematode-trapping fungi

Table 1 summarizes the sequence characterizations of newly determined subtilisin-like serine protease genes from five nematode-trapping fungi. The five sequences range from 1176-bp to 1288-bp in length and all contain an intron. The encoding genes of these five subtilisin-like serine proteases have been submitted to GenBank (Accession nos. EF113088 - EF113092).

**Table 1:** Basic information of cloned cuticle-degrading proteases from nematode-trapping fungi.

Gene	**Accession no**.	Species name	Whole/partial	Length (bp)	Intron (number)	Intron length (bp)	aa^a^	Molecular mass^b^
*Am1*	EF113088	*A. musiformis*	whole	1288	1	58	409	42 kDa
*Ay1*	EF113089	*A. yunnanensis*	whole	1283	1	52	409	42 kDa
*Mc1*	EF113090	*M. coelobrochum*	whole	1284	1	53	409	42 kDa
*Mp1*	EF113091	*M. psychrophilum*	partial	1176	1	54	373	--------
*Ds1*	EF113092	*D. shizishanna*	whole	1273	1	58	404	41.3 kDa

^a^, Number of amino acids in primary translation product.

^b^, Molecular mass was calculated online with ProtParam tools http://us.expasy.org/tools/protparam.html.

**Figure 1:**
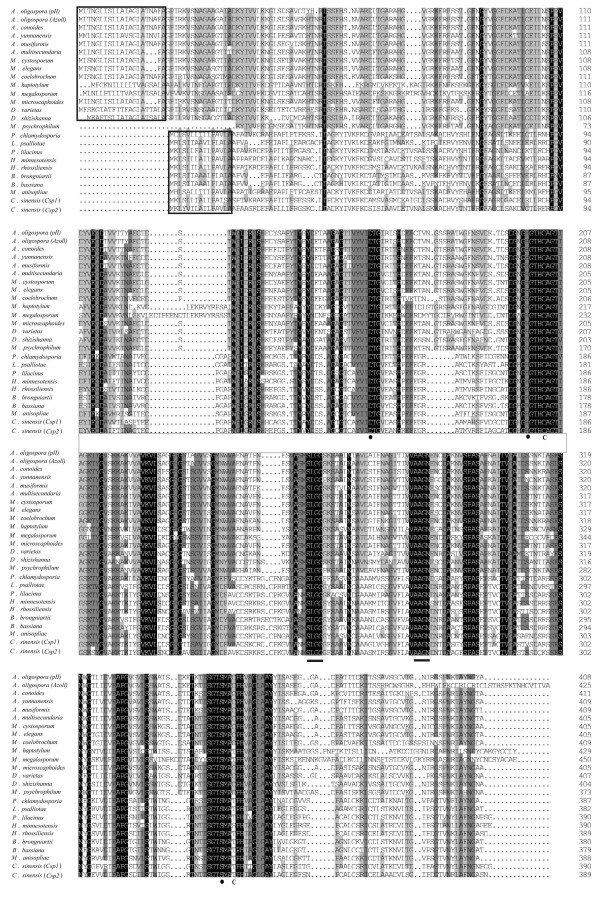
**Alignment of the cuticle-degrading protease sequences from nematophagous and entomopathogenic fungi**. Areas shaded in black are conserved regions (100% similarity), areas shaded in grey have a high degree of homology (more than 75% similarity) and unshaded areas are regions of variability between the proteases. Signal peptide sequences were encompassed by frame with black edge. • indicates the aspartic acid (Asp_41_)-histidine (His_77_)-serine (Ser_231_) (in EF113092) catalytic triad. C indicates the conserved cysteines. The underlined regions are the substrate-binding S_1 _pocket in subtilisin-like serine protease gene. GenBank number: *Arthrobotrys oligospora *(*PII*), X94121; *Arthrobotrys oligospora *(*Azol1*), AF516146; *Arthrobotrys conoides*, AY859782; *Arthrobotrys yunnanensi*s, EF113089; *Arthrobotrys musiformis*, EF113088; *Arthrobotrys multisecundaria*, EF055263; *Monacrosporium cystosporum*, AY859780; *Monacrosporium elegans*, AY859781; *Monacrosporium coelobrochum*, EF113091; *Monacrosporium haptotylum *EF681769; *Monacrosporium megalosporum*, AB120125; *Monacrosporium microscaphoide*s, AY841167; *Dactylella varietas*, DQ531603; *Dactylella shizishanna*, EF113092; *Monacrosporium psychrophilum*, EF113090;*Pochonia chlamydosporia*, AJ427460; *Lecanicillium psalliotae*, AY692148; *Paecilomyces lilacinus*, EF094858; *Hirsutella minnesotensis*, EF560594; *Hirsutella rhossiliensis*, DQ422145; *Beauveria brongniartii*, AY520814; *Beauveria bassiana*, EF195164;*Metarhizium anisopliae*, M73795; *Cordyceps sinensis *(*Csp1*), EU282382; *Cordyceps sinensis *(*Csp2*), EU282383.

Generally, the subtilisin-like serine proteases from nematode-trapping fungi, together with those from nematode-parasitic fungi and entomopathogenic fungi, were classified as cuticle-degrading proteases because they were involved in the penetration and digestion of nematode or insect cuticles [11-23]. Our analyses indicated that the subtilisin-like serine protease genes from nematode-trapping fungi shared a high degree of sequence similarity to those from nematode-parasitic fungi and entomopathogenic fungi. As seen from Figure 1, these subtilisin-like serine protease genes share a pre-pro-peptide structure. The signal peptides consisted of 15-21 amino acids. The potential catalytic triad residues (His, Asp and Ser) and the substrate-binding S_1 _pocket (Ser-Leu-Gly-Gly and Ala-Ala-Gly) were all conserved among these sequences. Moreover, two cysteines which contribute to the stability of the tertiary structure of proteases [24] were also conserved.

### Phylogenetic analyses

**Figure 2:**
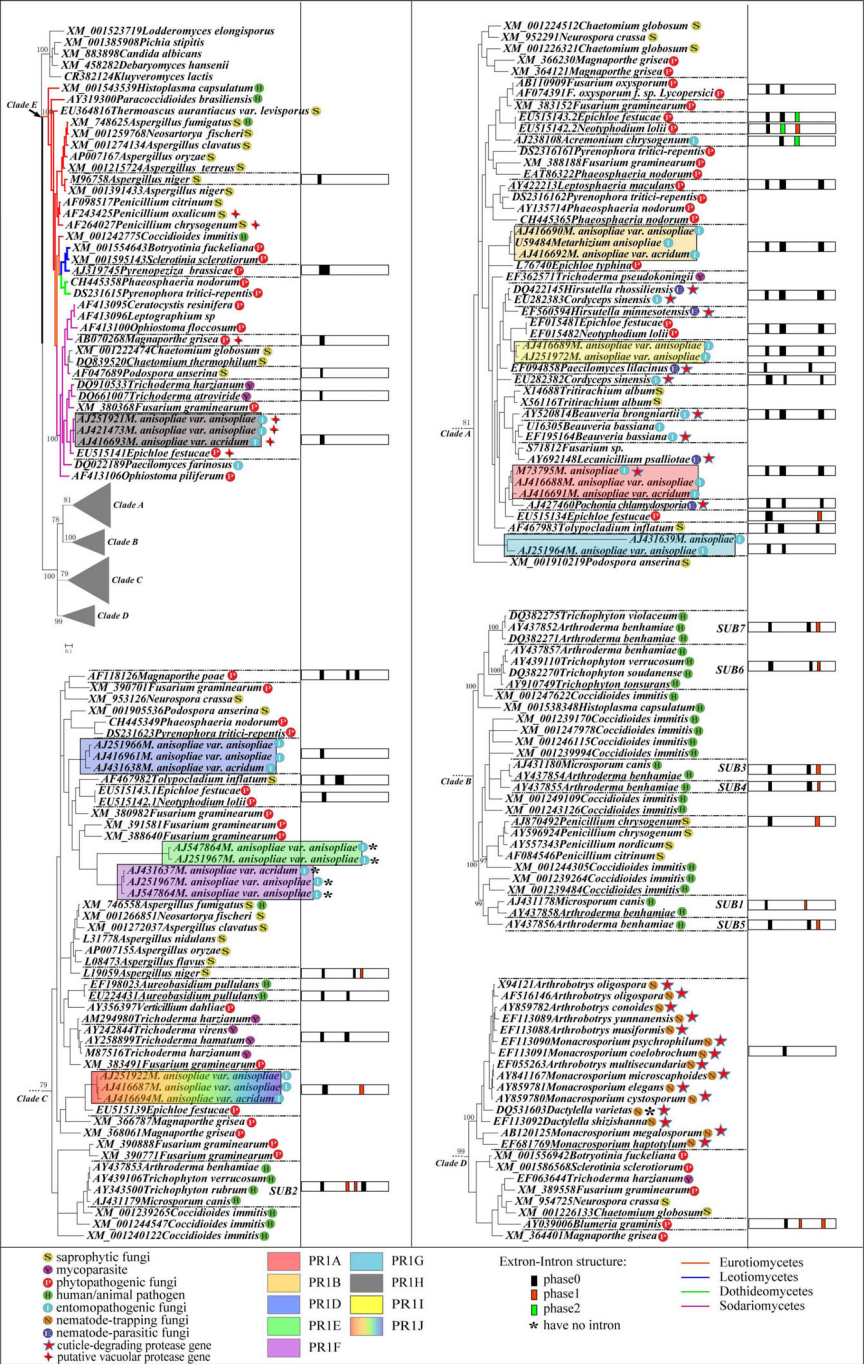
**Phylogenetic tree based on amino acid sequences of subtilisin-like serine protease genes**. The tree was constructed using MrBayes 3.1.2 [49]. The right frame diagrams show the intron distribution (position and phase) in corresponding proteins. SUB1-7 means seven genes encoding putative subtilisin-like serine proteases isolated from dermatophytic fungi.

Using various tree-building methods, our phylogenetic analyses based on a total of 189 subtilisin-like serine protease genes using various tree-building methods consistently showed five strongly-supported monophyletic clades. These clades are designated as A-E in the trees (Figure 2; Additional files 1, 2 and 3).

Our phylogenetic tree (Figure 2) clustered the cuticle-degrading protease genes from entomopathogenic and nematode-parasitic fungi into clade A with the exclusion of nematode-trapping fungi (BS = 81). The genes from nematode-trapping fungi all grouped into clade D (BS = 99). Clade B contained the subtilisin-like serine protease genes from fungi causing dermatosis in humans and animals. Clade E comprised sequences from four classes of Pezizomycotina (Sordariomycetes, Eurotiomycetes, Leotiomycetes and Dothideomycetes), as well as vacuolar protease genes [5,25-28], indicating the possible endocellular origin of these proteases.

Although the inconsistent relationships among the five clades were produced in our phylogenetic analyses, the earliest divergence of Clade E was strongly supported in all tree topologies (Figure 2; Additional files 1, 2 and 3). The alternative tree topologies, in which Clade E was not the basal clade in the phylogeny, were all found to be significantly worse than our tree (*P *< 0.05).

### Signatures of positive selection in the nematode-trapping fungi

**Figure 3:**
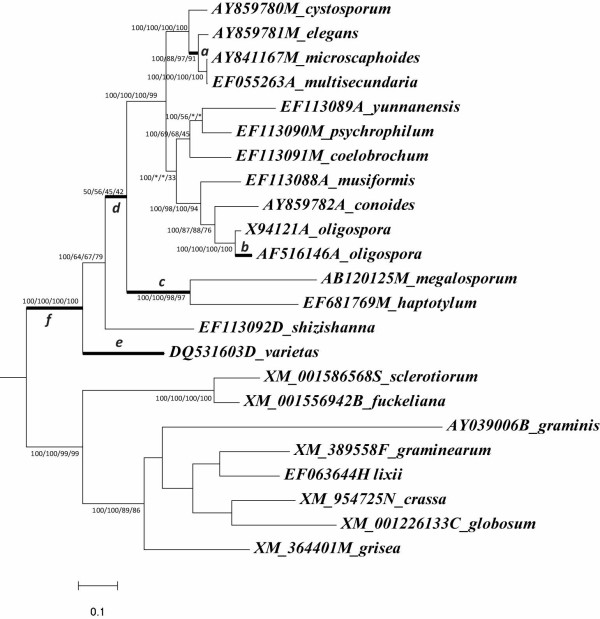
**Phylogeny based on subtilisin-like serine protease genes from nematode-trapping fungi used for ML analysis in PAML and Bn-Bs**. Maximum parsimony (MP), neighbor-joining (NJ), maximum-likelihood (ML) and Bayesian tree reconstructions of the subtilisin-like serine protease gene sequences of clade D presented similar overall topologies. The bootstrap values of each branch for different methodologies are indicated (Bayesian/ML/NJ/MP). The symbol (*) indicates distinct topological arrangements. The thick branches indicate the branch with a significant LRT in the PAML branch analysis. After calculation using Bn-Bs, only branch *f *(Z = 3.66320) was significant.

One of the intriguing findings in our study is evidence of positive selection in the nematode-trapping fungi. Maximum parsimony (MP), neighbor-joining (NJ), maximum-likelihood (ML) and Bayesian tree reconstructions of the subtilisin-like serine protease genes of nematode-trapping fungi presented similar overall topologies (Figure 3). Six branches (designated as *a-f*) showed signs of significant positive selection in all four tree topologies (Figure 3; Additional file 4). After Bonferroni correction for multiple testing, we found that likelihood ratio tests (LRT) were still significant in three branches (branch *b*, *e *and *f*) in all tree topologies (Figure 3; Additional file 4). Several positively selected residues were also identified for these branches with high posterior probabilities (see Additional file 4). Bonferroni correction in PAML analyses may be insufficient because this correction lacks statistical power [29]. We also measured the patterns of selection pressures among nematode-trapping fungal genes using the Bn-Bs program, a relatively conservative method. The analysis using the Bn-Bs program also revealed that *d*N was significantly greater than *d*S for branch *f*, the ancestor branch of nematode-trapping fungi (Z = 3.66320; Figure 3).

In sum, the results showed for the first time that positive selection might have acted on the subtilisin-like serine protease genes in nematode-trapping fungi, at least in the early stage of their evolution.

### Functional Bioassays

#### The infection of insects and nematodes with entomopathogenic fungi and nematode-parasitic fungi

One of the interesting results inferred from our phylogenetic analysis was the clustering of the cuticle-degrading protease genes from entomopathogenic and nematode-parasitic fungi. This result suggested that entomopathogenic fungi might have nematode-parasitic activities, and nematode-parasitic fungi might have entomopathogenic activities. To test this hypothesis, functional assays were conducted to determine whether the nematode-parasitic fungi could infect insects, and whether the entomopathogenic fungi could infect nematodes. Specifically, the entomopathogenic and nematode-parasitic fungi (Table 2) were tested on their potential to infect the eggs of both the root-knot nematode *Meloidogyne *sp. and the potato tuber moth *Phthorimaea opercullella*. The phytopathogenic fungus *Nigrospora oryzae *was used as the negative control.

**Table 2:** Fungal strains used in the bioassay.

YMF cultures (strain number)	Scientific Name	Pathogenicity	Reference(s)
	*Beauveria bassiana*^a^	Entomopathogenic	[13,67,68]
YMF1.00112	*Lecanicillium psalliotae*^b^	Nematophagous	[19,69,70]
	*Metarhizium anisopliae*^a^	Entomopathogenic	[71]
	*Metarhizium flavoviride*^c^	Entomopathogenic	[72,73]
	*Paecilomyces farinosus*^a^	Entomopathogenic	[74,75]
	*Paecilomyces fumosoroseus*^a^	Entomopathogenic	[76,77]
YMF1.00132	*Paecilomyces lilacinus*^b^	Nematophagous	[78,79]
YMF1.00130	*Pochonia chlamydosporia*^b^	Nematophagous	[80-82]
YMF5.00245	*Nigrospora oryzae*^b, d^	Phytopathogenic	[83]

^a^, Obtained from Prof. Zongqi Liang, Guiyang, Guizhou University, Guizhou Province, P. R. China.

^b^, Preserved in the Laboratory for Conservation and Utilization of Bio-resources, and Key Laboratory for Microbial Resources of the Ministry of Education, Yunnan University, Kunming, Yunnan Province, P. R. China.

^c^, Purchased from the Agricultural Culture Collection of China, Beijing, P. R. China.

^d^, Used as the negative control.

**Figure 4:**
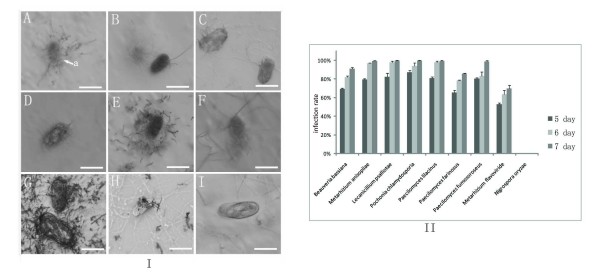
**The infection of the root-knot nematode *Meloidogyne *sp. with entomopathogenic fungi and nematode-parasitic fungi**. (I), Arrow with "a" was the eggs of the root-knot nematode *Meloidogyne *sp. A, *Beauveria bassiana*; B,*Metarhizium anisopliae*; C,*Lecanicillium psalliotae*; D,*Pochonia chlamydosporia*; E,*Paecilomyces lilacinus*; F,*Paecilomyces farinosus*; G,*Paecilomyces fumosoroseus*; H,*Metarhizium flavoviride*; I,*Nigrospora oryzae*. A-F, H and I, bar = 30 μm. G, bar = 15 μm. All the eight tested entomopathogenic or nematode-parasitic fungi (A-H) were able to infect the eggs of the root-knot nematode *Meloidogyne *sp. (II), The histogram shows the infection rate of 3 days. After 1 week, the infection rate of eight tested entomopathogenic or nematode-parasitic fungi ranged from 73% to 100%. The phytopathogenic fungus *N. oryzae *(I-I) that used as the negative control did not show any infection toward the eggs, so the infection rate was zero.

Our bioassay result on the eggs of the root-knot nematode *Meloidogyne *sp. indicated that all eight entomopathogenic or nematode-parasitic fungi could infect the eggs of the root-knot nematode (Figure 4). As seen from the histogram of Figure 4-II, the infection rate of these eight fungi ranged from 73% to 100% after 1 week, while the phytopathogenic fungus *N. oryzae *couldn't infect any egg.

**Figure 5:**
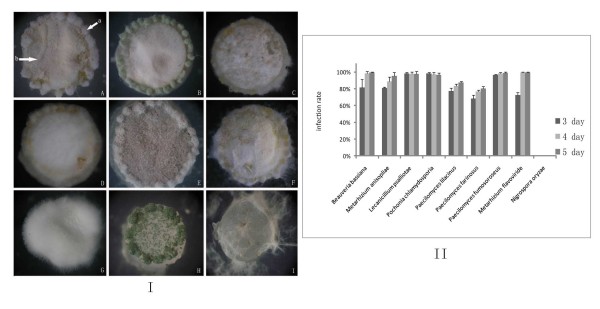
**The infection of the potato tuber moth *P. opercullella *with entomopathogenic fungi and nematode-parasitic fungi**. (I), Arrow with "a" was the eggs of potato tuber moth *P. opercullella*. Arrow with "b" was the selected fungal strains. Strains A-I have been described in Figure 4. Each fungal culture is 6 mm in diameter. For the negative control (I-I) showed that all of the juveniles had hatched within 1 week. (II), Although the bioassay lasted for 1 week, the infection rate up to highest after 5 days later, so we only show the infection rate of 3-5 days. The infection rate of eight tested entomopathogenic or nematode-parasitic fungi ranged from 80% to 100%. The phytopathogenic fungus *N. oryzae *(II-I) that used as the negative control did not show any infection toward the eggs and the juveniles were all hatched into juveniles within 5 days, so the infection rate was zero.

The bioassay on the eggs of *P. opercullella *indicated that all eight entomopathogenic or nematode-parasitic fungi could infect the eggs of the potato tuber moth (Figure 5). After 3 days of co-incubation, the juveniles hatched from the eggs. Five days later, the infection rates of the eight entomopathogenic or nematode-parasitic fungi were obtained and they ranged from 80% to 100% (Figure 5-II). For the negative control, all the potato tuber moth eggs hatched into juveniles within 5 days (Figure 5-I-I).

In sum, our bioassay results indicated that entomopathogenic and nematode-parasitic fungi could infect both nematode and insect eggs, thus supporting the inference from the phylogenetic analyses.

#### Effects of the subtilisin-like serine protease PSP-3 on the nematode and insect eggs

Subtilisin-like serine proteases are known to play a key role during penetration of nematode or insect cuticles [2,10]. In the study of Bonants et al., the subtilisin-like serine protease PSP-3 of nematode-parasitic fungus *Paecilomyces lilacinus *was found to be involved in the infection of *P. lilacinus *against the eggs of the root-knot nematode *Meloidogyne hapla *[30].

**Figure 6:**
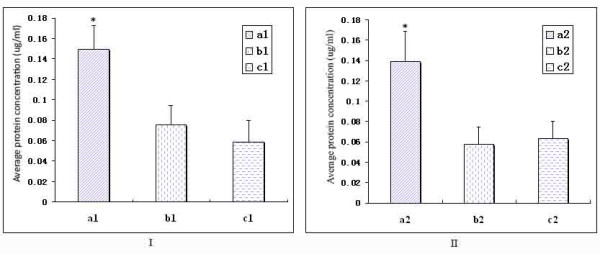
**Effects of the subtilisin-like serine protease PSP-3 on the nematode and insect eggs**. (I), Effect of the subtilisin-like serine protease PSP-3 produced by *P. lilacinus *on the eggs of the root-knot nematode *Meloidogyne *sp. The average amount of protein released into the supernatant of the treatment with the active protease (a1), none (b1) and denatured protease (c1); (*, *P *< 0.05). (II), Effect of the subtilisin-like serine protease PSP-3 produced by *P. lilacinus *on the eggs of the potato tuber moth *P. opercullella*. The average amount of protein released into the supernatant of the treatment with the active protease (a2), none (b2) and denatured protease (c2); (*, *P *< 0.05).

Based on our bioassay results, we hypothesize that the subtilisin-like serine proteases secreted by entomopathogenic or nematode-parasitic fungi should be able to digest both the nematode and insect eggs. We therefore purified PSP-3 from *P. lilacinus *to test its ability to digest the nematode and insect eggs. As expected, our bioassay results showed that the amount of protein released into the supernatant in the protease treatment group (treatment a) was significantly higher than those with no protease (treatment b) or denatured protease (treatment c) (Figure 6; Additional file 5). After incubation with active subtilisin-like serine proteases, the eggshell of the root-knot nematode and the potato tuber moth were partially degraded, and some eggs were deformed (data not show), suggesting that the subtilisin-like serine protease produced by *P. lilacinus *degraded protein components of both nematode and insect eggs.

## Discussion and Conclusions

Our study provides new insights into the evolution of subtilisin-like serine protease genes in Pezizomycotina. First, our phylogenetic results suggest that Pezizomycotina subtilisin-like serine proteases most likely evolved from endocellular to extracellular proteases. As seen from the tree, the subtilisin-like serine protease genes of Pezizomycotina previously identified or presumed to be endocellular proteases [5,25-28] were clustered into Clade E and diverged the earliest in the phylogeny. In contrast, most of those previously identified or presumed as extracellular proteases clustered into the other clades and diverged later. In addition, the protease (GenBank no. XM_001385908) from the outgroup *Pichia stipitis *was assumed as an endocellular protease [31], supporting the endocellular to extracellular transition during the evolution of Pezizomycotina subtilisin-like serine proteases. The evolution from endocellular to extracellular enzymes might have facilitated pathogenic fungi to utilize these enzymes as virulence factor to colonize their hosts.

Second, our phylogenetic results and functional bioassays suggested that the entomopathogenic and nematode-parasitic fungi shared similar properties in parasitism. Previous studies indicated that the nematode-parasitic fungi could infect nematodes while the entomopathogenic fungi could parasitize other insects [8,32]. The grouping of the cuticle-degrading protease genes of these two groups of fungi led us to believe that they might have overlapping pathogenic mechanisms against both insects and nematodes. Further experimental bioassay showing that the entomopathogenic and nematode-parasitic fungi could infect both nematode and insect eggs supported this hypothesis (Figure 4 and 5). Moreover, the subtilisin-like serine protease PSP-3 produced by nematode-parasitic fungus *P. lilacinus *was observed to have the ability to digest the eggs of nematodes and insects (Figure 6; Additional file 5). Our finding enriches the known fungal resources for the microbial control of both types of pests.

Third, our data provide further understanding about the duplications and subsequent functional divergence of subtilisin-like serine protease genes in Pezizomycotina. Previous studies have shown that gene duplications occurred in subtilisin-like serine protease genes of pathogenic fungi and that such duplications might have played an essential role in pathogenicity and contributed to their increased adaptability, host range, and/or survived in various ecological habitats outside the host of these fungi [33].

From our phylogenetic tree, we found that frequent gene duplication events have occurred in many pathogenic fungi. For example, there were 9 paralogous subtilisin-like serine protease genes in *Metarhizium anisopliae*. Four of them, including *PR1A*, *PR1B*, *PR1I *and *PR1G*, clustered into Clade A with the other cuticle-degrading protease genes known to serve as important virulence factors during fungal penetration of nematode or insect cuticles [13-17,19]. These four genes most likely perform essential functions during the infection of hosts. In comparison, *PR1J*, *PR1D*, *PR1E *and *PR1F *of *M. anisopliae *fell into Clade C, indicating that they might have diverged to perform different as yet uncharacterized functions. Our results are different from previous inferences suggesting that *PR1A *was a key virulence factor during degradation of insect cuticles, while all the other *PR1*'s were of only a minor contributor to cuticle degradation [25,34].

In the dermatophytic fungus *Arthroderma benhamiae*, there are 7 subtilisin-like serine protease genes (SUB1-7) [35]. Our phylogenetic tree clustered 6 of them in Clade B. The only exception was SUB2, which is grouped into Clade C. This result is consistent with the phylogeny of Jousson et al. [35]. They hypothesized that SUB2 was the most ancestral, while the other SUBs were dermatophyte-specific and might have emerged more recently through successive gene duplication events. In addition, they speculated that SUB3 and SUB4 were key virulence proteases of dermatophytes and playing important role in invasion of human or animal keratinised tissues [35]. From our analysis, the key virulence protease genes of dermatophytic fungi and nematophagous/entomopathogenic fungi were placed in different clades, suggesting that there were probably different pathogenic mechanisms between mammalian pathogens and nematode/insect pathogens.

Fourth, our evolutionary analysis revealed signatures of positive selection acting on the subtilisin-like serine protease genes of the nematode-trapping fungi, suggesting that the subtilisin-like serine protease genes might have played important roles during the evolution of pathogenicity of nematode-trapping fungi against nematodes. Although they both belonged to the cuticle-degrading proteases, the subtilisin-like serine protease genes from nematode-trapping fungi and nematode-parasitic fungi were not grouped together. This result supports earlier findings based on smaller data sets [10,11,17]. Since nematode-parasitic fungi do not produce trapping devices, they likely rely mainly on the extracellular enzymes (including subtilisin-like serine protease) as virulence factors to penetrate and digest nematode cuticles. In contrast, by virtue of synergistic interactions of extracellular enzymes and trapping devices, the nematode-trapping fungi can immobilize and penetrate nematodes within a few hours [2,10]. Therefore, the subtilisin-like serine proteases of nematode-trapping fungi might experience special selective pressure resulted from the co-evolution of trapping structures and proteolytic enzymes. Interestingly, our evolutionary analysis demonstrated evidence of positive selection acting on the cuticle-degrading protease genes in nematode-trapping fungi, at least during the early stage of its evolution. We hypothesize that the subtilisin-like serine protease genes may have played important roles during the evolution of pathogenicity of nematode-trapping fungi against nematodes. In addition, the potentially adaptive amino acid replacements discovered by our analysis will provide valuable information for functional analysis in future studies.

## Methods

### Samples collection

The five nematode-trapping fungi (*Arthrobotrys yunnanensis *YMF1.00593, *Arthrobotrys musiformis *YMF1.01043, *Monacrosporium psychrophilum *YMF1.01412, *Monacrosporium coelobrochum *YMF1.01480 and *Dactylella shizishanna *YMF1.00022) were isolated from field soil samples in Yunnan Province of China and permanently stored in the Yunnan Microbiological Fermentation Culture Collection Center (YMF). They were maintained on cornmeal agar (CMA) at 28°C.

### Genomic DNA extraction

The five nematode-trapping fungi were cultured in the PL-4 liquid medium on a rotary shaker (150 rpm) at 28°C for 1 week [36]. Their mycelia were then filtered on a nylon mesh and genomic DNA was isolated using the E.Z.N.A.^@ ^Fungal DNA Mini kits (Omega Bio-Tek, Inc. USA) following the manufacturer's protocol.

### Primers design and cloning of subtilisin-like serine protease genes

Degenerate primers (NP, NR, and P246) (Table 3) were designed based on previously reported six subtilisin-like serine protease genes from nematode-trapping fungi *Arthrobotrys oligospora *(GenBank no. X94121), *Arthrobotrys conoides *(GenBank no. AY859782), *Monacrosporium microscaphoides *(GenBank no. AY841167), *Monacrosporium cystosporum *(GenBank no. AY859780), *Monacrosporium elegans *(GenBank no. AY859781) and *Dactylella varietas *(GenBank no. DQ531603). NP and NR were used to amplify the full-length genes of subtilisin-like serine proteases from *A. musiformis *and *A. yunnanensis*, while P246 and NR were used to amplify the 3'-end fragment of the genes from *M. coelobrochum*, *M. psychrophilum *and *D. shizishanna*. The primers 1480TSPN (N = 1, 2, 3) and 22TSPN (Table 3), which were designed according to the amplified sequences of serine proteases, were respectively used to amplify the 5'-end sequences from *M. coelobrochum *and *D. shizishanna *by using the DNA Walking Speedup™ PreMix Kit (Seegene, Korea) according to the user's manual.

**Table 3:** List of primers used in this study.

Primer	Primer Sequence
NP	5-AATG(A/C)T(G/T)(A/T)(C/T)GAACGGCCT(C/T)A-3
NR	5-TTAAGC(G/A)(G/T)(A/T/C)(G/T)CC(G/A)TTGTAG-3
P246	5-AA(G/A)TA(C/T)AT(C/T)GTCGTC(C/T)(A/T)(C/G)AAG-3
1480TSP1	5-ATGAGAGATGCGGTCAAGGC-3
1480TSP2	5-CGACTTCAGGCGAGTTCAGAATC-3
1480TSP3	5-CAAAGCCGCCAGTGTATCCAG-3
22TSP1	5-TGAGAGATGCGGTCAAGAC-3
22TSP2	5-TATCCTGAAGAGTAGCAGCGTCG-3
22TSP3	5-GGCGTGGAGAGATGAAATGCG-3

NP and NR were used to amplify the full-length encoding genes of subtilisin-like serine proteases from *A. musiformis *and *A. yunnanensis*. P246 and NR were used to amplify the 3' fragment of the subtilisin-like serine protease genes from *M. coelobrochum*, *M. psychrophilum *and *D. shizishanna*. 1480TSPN (N = 1, 2, 3) and 22TSPN (N = 1, 2, 3) were used to amplify the 5' fragment sequences from *M. coelobrochum *and *D. shizishanna *by using the DNA Walking Speedup™ PreMix Kit (Seegene, Korea), respectively

The PCR reaction mixture was consisted of 0.5 μL Taq DNA polymerase, 5 μL of reaction mixture buffer, 3 μL of 25 mM MgCl_2_, 1 μL of 2.5 mM dNTPs, 1 μL of 100 μM degenerate primers, 3 μL of DNA template in a final volume of 50 μL supplied with double-distilled sterile water. Amplification started at 95°C for 5 min, followed by 35 cycles with 95°C for 40 s, 51°C for 40 s, and 72°C for 1.5 min. After the last cycle, the reaction mixture was maintained at 72°C for 10 min for a final extension step.

### Sequencing and sequence analysis

The amplified products were electrophoresed on 1% agarose gels to check for fragment size and purity. All the PCR products were purified using the DNA fragment purification kit ver 2.0 (Takara, Japan) and sub-cloned into the vector pMD18-T (Takara, Japan). *Escherichia coli *strain DH5α was used as a host for transformation and cloning. It was grown in Luria-Bertani medium at 37°C. Ten positive colonies were selected for sequencing from each strain. The plasmids were sequenced in both directions on an ABI 3730 automated sequencer (Perkin-Elmer, USA). Sequence assembly was performed using the SeqMan software (DNA Star software package, DNASTAR, Inc. USA) and DNAman software package (Version 5.2.2, Lynnon Biosoft, Canada). Signal sequence was predicted using SignalP [37]http://www.cbs.dtu.dk/services/SignalP/. Protein molecular masses were determined online with ProtParam tools http://us.expasy.org/tools/protparam.html, and N-linked glycosylation sites were predicted by NetNGlyc http://www.cbs.dtu.dk/services/NetNGlyc/.

### Subtilisin-like serine protease genes from the other Pezizomycotina species

The amino acid sequences of each protease characterized as members of the subtilisin-like family were retrieved from National Center for Biotechnology Information (NCBI) [38] using BLASTX [39]. MEROPS and UniProtKB (UniProt Knowledgebase) protein sequence database were also screened to iteratively search all known and predicted subtilisin-like serine protease genes, making sure that all available subtilisin-like serine protease genes of Pezizomycotina were included in the analyses. A total of nearly 500 subtilisin-like serine protease genes representing five of nine classes in Pezizomycotina, including Sordariomycetes, Eurotiomycetes, Leotiomycetes, Dothideomycetes and Orbiliomycetes, were obtained. In addition, five subtilisin-like serine protease genes from fungi in the Saccharomycotina were downloaded and used as outgroups. The partial sequences and those with unusual lengths (<200 aa or >700 aa) in Pezizomycotina were removed from the analysis, yielding 287 sequences.

The amino acids sequences of the 287 genes were aligned using Clustal X version 1.83 [40] with default parameters. Several sequences with ambiguously aligned regions around the three active catalytic residues (Asp-His-Ser) and those with identical sequences but different accession numbers were eliminated in the analysis, yielding 189 subtilisin-like sequences in the final dataset. The Gblocks program http://molevol.cmima.csic.es/castresana/Gblocks.html[41,42] was applied to extract conserved regions that contain more reliable phylogenetic signals. An alignment consisting of 229 amino acid positions was obtained.

### Phylogenetic analysis

Four tree-building methods were performed for phylogenetic reconstructions. The maximum parsimony (MP) tree with heuristic search was constructed using PAUP*4.0b8 [43], and maximum likelihood (ML) tree with the best-fit model (WAG+I+G) was constructed using PHYML version 2.4.4 [44]. The best-fit model of protein evolution was selected by ProtTest http://darwin.uvigo.es[45]. In addition, the program MEGA 4.1 [46,47] was used to construct a neighbor joining (NJ) tree [48] and MrBayes 3.1.2 [49] was used to perform Bayesian analysis. Bayesian analysis started with randomly generated trees and four Markov chains under default heating values were ran for 2 × 10^6 ^generations, with sampling at intervals of 100 generations. To ensure that these analyses were not trapped in local optima, the dataset was run three times independently. We determined the burn-in period by checking for likelihood stability.

To test for nodal reliabilities, bootstrap (BS) analysis [50] for MP (1,000 replicates), ML (100 replicates) and NJ (1,000 replicates) was applied. Bayesian posterior probabilities (PP) from the 50% majority-rule consensus tree were calculated to provide the estimates of nodal support in Bayesian phylogenies.

### Testing the inconsistency of tree topologies

AU-test [51], SH-test [52] and KH-test [53] were performed using CONSEL version 0.1 h [54] with site-wise log-likelihoods calculated by Tree-Puzzle 5.1 [55] to assess the inconsistency of tree topologies.

### Detecting positive selection in nematode-trapping fungi

Positive selection has been considered a major force in forming new motifs or functions in proteins [56]. The non-synonymous to synonymous rate ratio ω (*d*N/*d*S) provides an indication of the change in selective pressures. A *d*N/*d*S ratio = 1, <1, and >1 indicates neutral evolution, purifying selection, and positive selection on the protein involved, respectively. In this study, CODEML program of the PAML package [57,58] and the Bn-Bs software http://www.bio.psu.edu/People/Faculty/Nei/Lab/software.htm[59] were implemented to detect signatures of positive selection in nematode-trapping fungi. The multiply aligned data are available as supplementary material (see Additional file 6).

The Bn-Bs software implements a modified method from the original [60] by taking into account the transition bias for estimating synonymous and nonsynonymous substitutions along the branches of a given tree. Here, one-tailed Z test was performed.

Considering that positive selection may act in very short episodes during the evolution of a protein [61] and affect only a few sites along a few lineages in the phylogeny, the "branch-site" model, which allows ω ratios to vary both among lineages of interest and amino acid sites, was considered here in codon-based likelihood analysis using the PAML software [57,58]. We used the branch-site Model A as a stringent test to identify the sites under positive selection along the lineages of interest [62] and each model was run twice. All the possible tree topologies were used here.

### The infection of insects and nematodes with entomopathogenic fungi and nematode-parasitic fungi

For bioassays against the root-knot nematode, nine selected fungi (Table 2) were cultivated in 9 sterile Water Agar (WA) media (15 g agar in 1 L deionized water) at room temperature (10-25°C) separately, until each agar medium surface was colonized by the fungus. Then a suspension of nematode eggs (*ca*. 400 eggs) was added onto the fungal culture and the mixture was then kept at the room temperature. In order to keep the eggs moisturized, a 200 μL sterile M9 buffer (Na_2_HPO_4 _6 g, KH_2_PO_4 _3 g, NaCl 0.5 g, NH_4_Cl 1 g, deionized water to 1 L) was added daily. After 5 days, each culture was observed daily with the Olympus BX51 microscope (Japan) and counted the infected eggs 3 times each day. The criterion of infection was that the root-knot nematode eggs were failed to hatch and the fungal mycelia were seen growing from the eggs, while none could be seen in the negative control. All assays were repeated 3 times and each replicates lasted a week.

For bioassays against the potato tuber moth, a fungal culture with 6 mm in diameter was inoculated onto the center of a WA medium. Then exactly 30 insect eggs were put on the periphery of the fungal culture and incubated at room temperature. From 3 days onward, each culture was observed daily and counted the infected eggs 3 times each day. The criterion of infection was that the potato tuber moth eggs were failed to hatch and the fungal mycelia were seen growing from the eggs. All assays were repeated 3 times and each replicate lasted for 1 week.

### Effects of the subtilisin-like serine protease PSP-3 on the nematode and insect eggs

The subtilisin-like serine protease PSP-3 produced by the nematophagous fungus *P. lilacinus *was tested for its ability to degrade the eggs of the root-knot nematode *Meloidogyne *sp. and potato tuber moth *P. opercullella*. The protease PSP-3 was purified following the procedures described by Bonants et al. [30].

The following experiment design was used: (treatment a). nematode (a1) or insect (a2) eggs, protease (1,000 μg/mL, 0.2 U/mL, dissolved in potassium phosphate buffer) 10 μL, toluene (as an antiseptic) 3 μL, potassium phosphate buffer (0.1 mol/L) 137 μL; (treatment b). nematode (b1) or insect (b2) eggs, toluene 3 μL, potassium phosphate buffer (0.1 mol/L) 147 μL; (treatment c). nematode (c1) or insect (c2) eggs, denatured protease (1,000 μg/mL, 0.2 U/mL, dissolved in potassium phosphate buffer and then denatured by boiling for 30 min) 10 μL, toluene 3 μL, potassium phosphate buffer (0.1 mol/L) 137 μL; and (d). toluene 3 μL, potassium phosphate buffer (0.1 mol/L) 147 μL. Protein concentration was measured using Coomassie Brilliant Blue G-250 according to Bradford [63], with bovine serum albumin (25-400 μg/mL) as the standard. One unit of protease was defined as the amount of enzyme that released 1 mg tyrosine/min at 25°C. For the nematode assay, *ca*. 1700 eggs were used in each treatment. For the insect assay, exactly 30 eggs were used. All the treatments were shaken at Speed 3 on the mixer (Cole-Parmer, USA) at 25°C. Every 24 h, each treatment was precipitated and a 20 μL- supernatant was collected to measure the amount of protein concentration using casein as substrate. The supernatant from treatment (d) was used as the negative control. Whole assay was repeated 3 times. After checking the data with the Kolmogorov-Smirnov test and Shapiro-Wilk test [64-66], we found the data from each treatment are approximated a normal distribution (*P *≥ 0.05) (see Additional file 7). Statistics analyses were then carried out using one-way analysis of variance (ANOVA) in SPSS 14.0. Moreover, the LSD-test was also used for pairwise comparisons among the three treatments (see Additional file 5). A *P *value of less than 0.05 was considered as statistically significant.

## Authors' contributions

JL, LY, JY, and KZ conceived this study. JL and LD conducted all experimental work. JL collected data, carried out analyses and wrote the draft manuscript. LY and BT contributed to data analyses. JY and LY contributed to manuscript revisions. All other authors helped in interpretation of data and discussion of results. All authors read and approved the manuscript.

## Supplementary Material

Additional file 1 **MP tree**. The MP tree with heuristic search was constructed using PAUP*4.0b8 [43] with 1,000 replicates. Click here for file

Additional file 2 **NJ tree**. The program MEGA 4.1[46,47] was used to construct a neighbor joining (NJ) tree with 1,000 replicates. Click here for file

Additional file 3 **ML tree**. The ML tree with the best-fit model (WAG+I+G) was constructed using PHYML version 2.4.4 [44]. The best-fit model of protein evolution was selected by ProtTest http://darwin.uvigo.es[45]. Click here for file

Additional file 4 **Evidence of adaptive evolution from branch-site model analysis for cuticle-degrading protease genes from nematode-trapping fungi**. The branch-site model analysis using CODEML program of the PAML package [57,58] was used to detect signatures of positive selection in the cuticle-degrading proteases from nematode-trapping fungi. Six branches (designated as *a-f*) showed signs of significant positive selection in all four tree topologies. Several positively selected residues were also identified for these branches with high posterior probabilities. ^a^, After Bonferroni correction for multiple testing, branches *b*, *e *and *f *are still significant of all four tree topologies. ^b^, In *L *is the log-likelihood scores. ^c^, LRT to detect adaptive evolution. *** *P *< 0.001; **0.001 <*P *< 0.01; * 0.01 <*P *< 0.05.^d^, Those codons with posterior probabilities >99% are shown in boldface. Click here for file

Additional file 5 **Results of one-way analysis of variance (ANOVA) and LSD-test using SPSS 14.0**. The data of each treatment were carried out using one-way analysis of variance (ANOVA) in SPSS 14.0. Moreover, the LSD-test was also used for pairwise comparisons among the three treatments. A *P *value of less than 0.05 was considered as statistically significant. The results showed that the average amount of protein released into the supernatant in the protease treatment group (treatment a) was significantly higher than those with no protease (treatment b) or denatured protease (treatment c). I^a^, The subtilisin-like serine protease PSP-3 produced by *P. lilacinus *on the eggs of the root-knot nematode *Meloidogyne *sp. II^b^, The subtilisin-like serine protease PSP-3 produced by *P. lilacinus *on the eggs of the potato tuber moth *P. opercullella*. Click here for file

Additional file 6 **Multiple nucleotide alignment results used to do positive selection analysis**. The nucleotide sequences were translated to amino acids and aligned using MUSCLE Version 3.7 [84]. The alignment was then used to extract the conserved alignment regions using the Gblocks program http://molevol.cmima.csic.es/castresana/Gblocks.html[40,41]. Finally, 792 conserved nucleotide sites were obtained. Click here for file

Additional file 7 **Detection of normal distribution with the Kolmogorov-Smirnov test and Shapiro-Wilk test in SPSS 14.0**. The 18 data points of each treatment were checked for normal distribution with Kolmogorov-Smirnov test and Shapiro-Wilk test [64-66] in SPSS 14.0. The results showed that the data from the three treatments all approximated a normal distribution (*P *≥ 0.05). I^a^, The subtilisin-like serine protease PSP-3 produced by *P. lilacinus *on the eggs of the root-knot nematode *Meloidogyne *sp. II^b^, The subtilisin-like serine protease PSP-3 produced by *P. lilacinus *on the eggs of the potato tuber moth *P. opercullella*. Click here for file
