# Supplementary figures and images for: New insights into the evolution of subtilisin-like serine protease genes in Pezizomycotina
Source: BMC Evol Biol. 2010 Mar 9;10:68. doi: 10.1186/1471-2148-10-68 (PMC2848655; doi:10.1186/1471-2148-10-68)

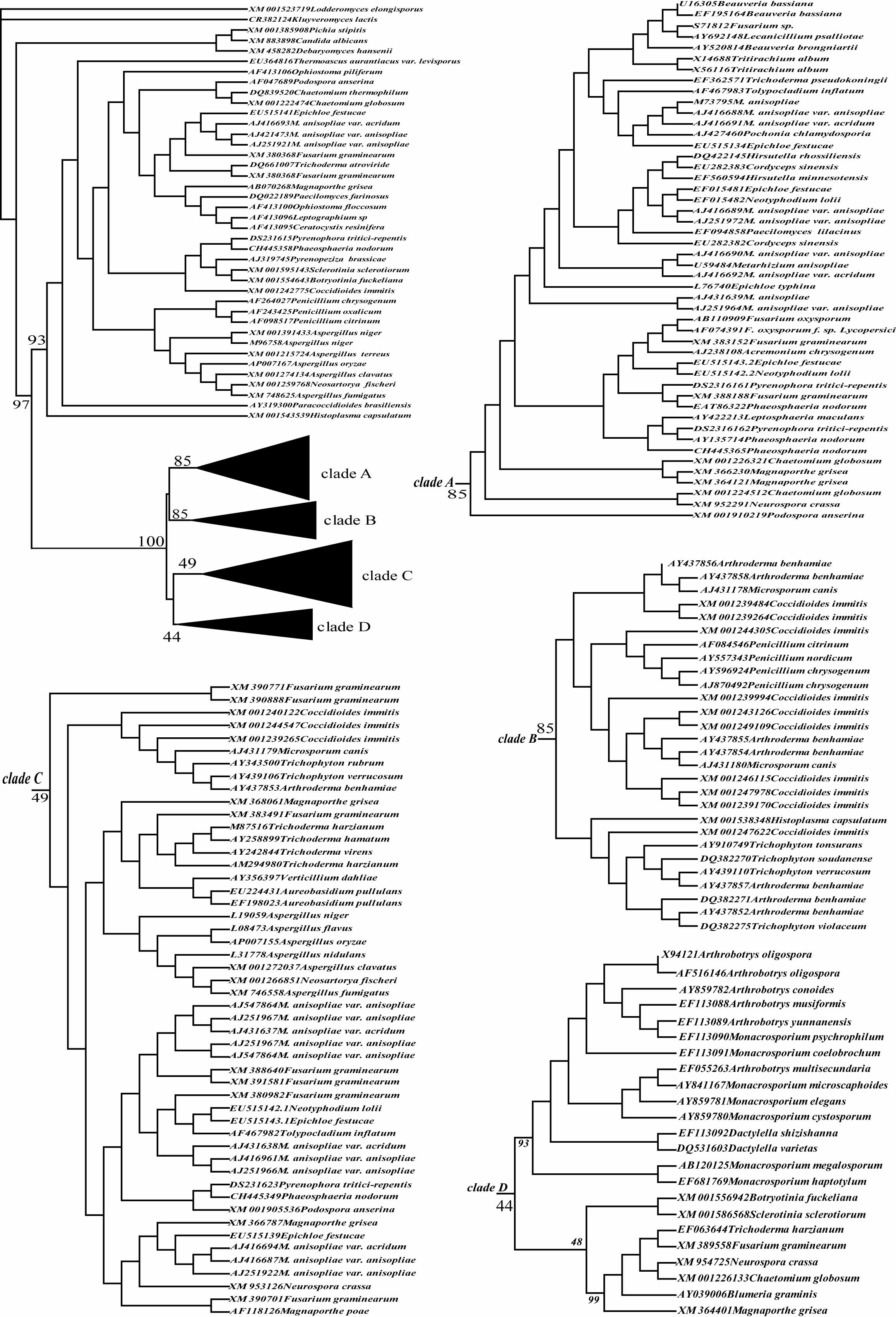

Supplement: Additional file 1 — MP tree. The MP tree with heuristic search was constructed using PAUP*4.0b8 [43] with 1,000 replicates. [file 1471-2148-10-68-S1.JPEG]

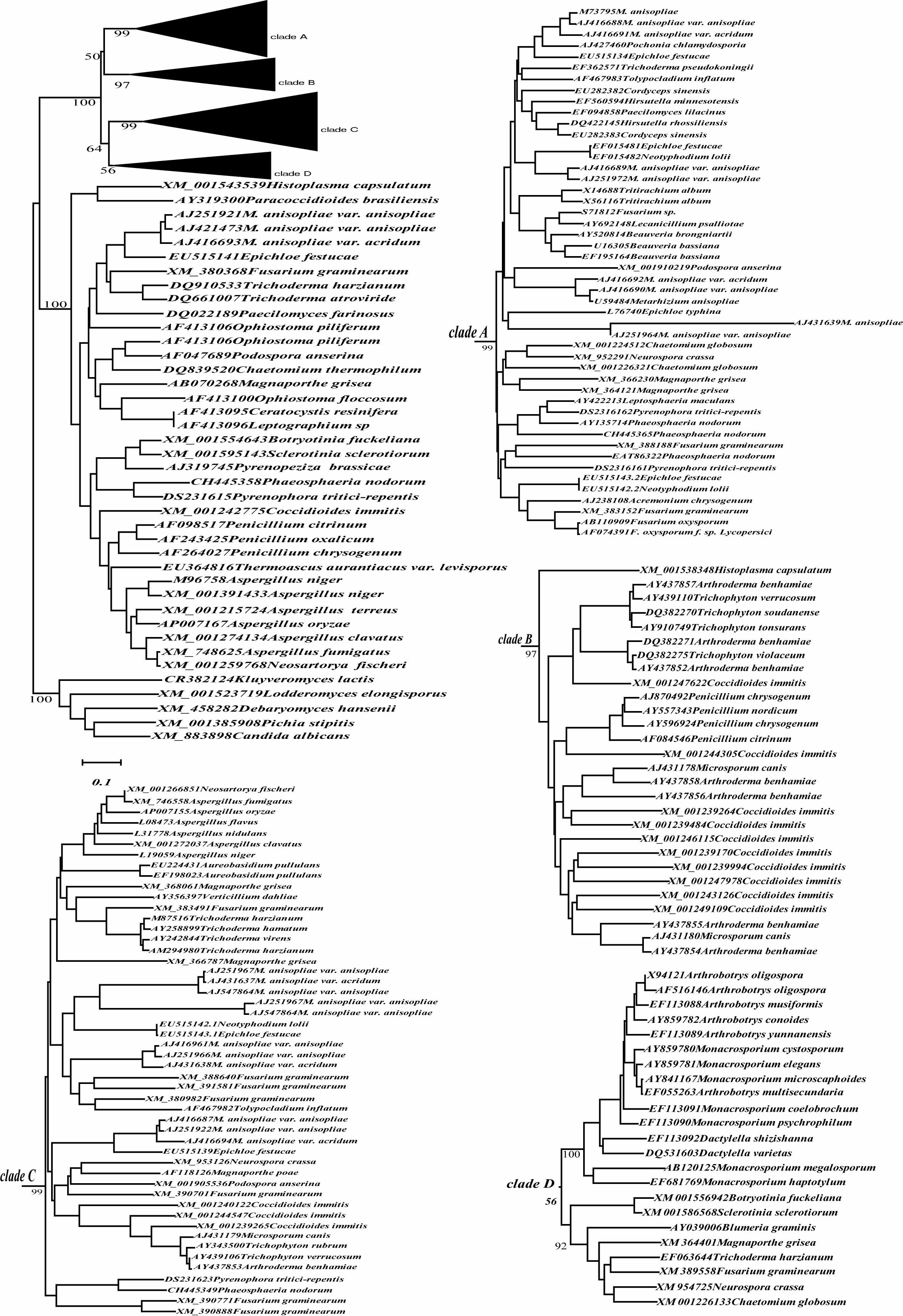

Supplement: Additional file 2 — NJ tree. The program MEGA 4.1[46,47] was used to construct a neighbor joining (NJ) tree with 1,000 replicates. [file 1471-2148-10-68-S2.JPEG]

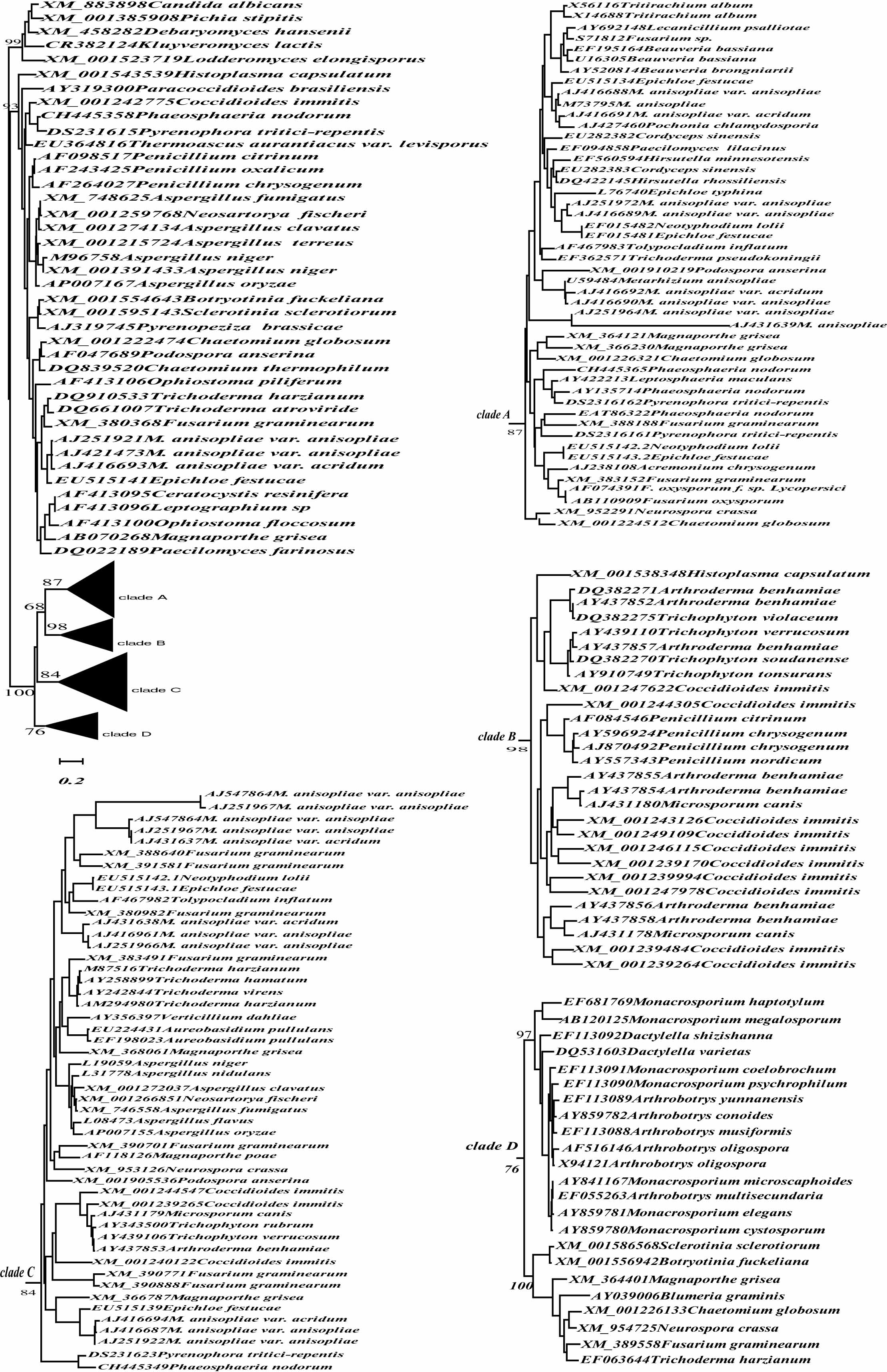

Supplement: Additional file 3 — ML tree. The ML tree with the best-fit model (WAG+I+G) was constructed using PHYML version 2.4.4 [44]. The best-fit model of protein evolution was selected by ProtTest http://darwin.uvigo.es[45]. [file 1471-2148-10-68-S3.JPEG]
