# Supplementary material for: New insights into the evolution of subtilisin-like serine protease genes in Pezizomycotina
Source: BMC Evol Biol. 2010 Mar 9;10:68. doi: 10.1186/1471-2148-10-68 (PMC2848655; doi:10.1186/1471-2148-10-68)
Supplement: Additional file 4 — Evidence of adaptive evolution from branch-site model analysis for cuticle-degrading protease genes from nematode-trapping fungi. The branch-site model analysis using CODEML program of the PAML package [57,58] was used to detect signatures of positive selection in the cuticle-degrading proteases from nematode-trapping fungi. Six branches (designated as a-f) showed signs of significant positive selection in all four tree topologies. Several positively selected residues were also identified for these branches with high posterior probabilities. a, After Bonferroni correction for multiple testing, branches b, e and f are still significant of all four tree topologies. b, In L is the log-likelihood scores. c, LRT to detect adaptive evolution. *** P < 0.001; **0.001 <P < 0.01; * 0.01 <P < 0.05.d, Those codons with posterior probabilities >99% are shown in boldface. [file 1471-2148-10-68-S4.DOCX]

**Additional file 4-Evidence of adaptive evolution from branch-site model analysis for cuticle-degrading proteases from nematode-trapping fungi.**

| Branch-site models*^a^* | Phylogeny |  | In*L^b^* | Parameter Estimates | 2Δ*L^c^* | Positively Selected Sites*^d^* |
| --- | --- | --- | --- | --- | --- | --- |
| Branch *a* | Bayesian | model A | -11635.42198 | ω0=0.08218,ω1=1,ω2=1,p0=0.57960,p2a=0.04498,p2b=0.07517 | 8.284812* | ***104*** |
|  |  | M1a | -11639.56439 | ω0=0.08351,ω1=1,ω2=1,p0=0.70023,p2a=0.00000,p2b=0.00000 |  |  |
|  | ML | model A | -11640.17367 | ω0=0.08199,ω1=1,ω2=1,p0=0.58791,p2a=0.11250,p2b=0.04812 | 15.589946*** |  |
|  |  | M1a | -11632.3787 | ω0=00.08196,ω1=1,ω2=169.18894,p0=0.69139,p2a=0.00789,p2b=0.00339 |  |  |
|  | MP | model A | -11642.57843 | ω0=0.08473,ω1=1,ω2=1,p0=0.70454,p2a=0.00000,p2b=0.00000 | 23.277504*** |  |
|  |  | M1a | -11630.93967 | ω0=0.08335,ω1=1,ω2=145.49807,p0=0.69404,p2a=0.00805,p2b=0.00341 |  |  |
|  | NJ | model A | -11645.37685 | ω0=0.08218,ω1=1,ω2=1,p0=0.58326,p2a=0.12196,p2b=0.05098 | 13.950856*** |  |
|  |  | M1a | -11638.40142 | ω0=0.08233,ω1=1,ω2=155.17630,p0=0.69803,p2a=0.00679,p2b=0.00284 |  |  |
| Branch *b* | Bayesian | model A | -11624.23504 | ω0=0.07883,ω1=1,ω2=1,p0=0.47749,p2a=0.21494,p2b=0.09547 | 75.277256*** | *229,* ***252,****253, 254, 256,* ***257****, 258,* ***259****,* ***262****,* ***263****, 264* |
|  |  | M1a | -11586.59641 | ω0=0.07830,ω1=1,ω2=∞,p0=0.64251,p2a=0.04498,p2b=0.02045 |  |  |
|  | ML | model A | -11628.84782 | ω0=0.07852,ω1=1,ω2=1,p0=0.47768,p2a=0.21533,p2b=0.09539 | 75.478428*** |  |
|  |  | M1a | -11591.10861 | ω0=0.07797,ω1=1,ω2=∞,p0=0.64275,p2a=0.04496,p2b=0.02042 |  |  |
|  | MP | model A | -11627.52000 | ω0=0.07998,ω1=1,ω2=1,p0=0.48341,p2a=0.21149,p2b=0.09286 | 74.758148*** |  |
|  |  | M1a | -11590.14093 | ω0=0.07932,ω1=1,ω2=∞,p0=0.64417,p2a=0.04509,p2b=0.02033 |  |  |
|  | NJ | model A | -11634.42944 | ω0=0.07884,ω1=1,ω2=1,p0=0.48638,p2a=0.21131,p2b=0.09156 | 74.99097*** |  |
|  |  | M1a | -11596.93395 | ω0=0.07820,ω1=1,ω2=∞,p0=0.64681,p2a=0.04518,p2b=0.02011 |  |  |
| Branch *c* | Bayesian | model A | -11632.9232 | ω0=0.08016,ω1=1,ω2=1,p0=0.58178,p2a=0.11729,p2b=0.05049 | 13.857264*** | ***133, 152*** |
|  |  | M1a | -11625.99457 | ω0=0.08065,ω1=1,ω2=104.84592,p0=0.64915,p2a=0.05200,p2b=0.02216 |  |  |
|  | ML | model A | -11637.24307 | ω0=0.07972,ω1=1,ω2=1,p0=0.57419,p2a=0.12568,p2b=0.05390 | 11.096814** |  |
|  |  | M1a | -11631.69466 | ω0=0.08023,ω1=1,ω2=81.87326,p0=0.65140,p2a=0.05091,p2b=0.02158 |  |  |
|  | MP | model A | -11636.06556 | ω0=0.08140,ω1=1,ω2=1,p0=0.58448,p2a=0.11741,p2b=0.04987 | 13.39042** |  |
|  |  | M1a | -11629.37035 | ω0=0.08195,ω1=1,ω2=109.56799,p0=0.65203,p2a=0.05181,p2b=0.02180 |  |  |
|  | NJ | model A | -11642.51764 | ω0=0.07994,ω1=1,ω2=1,p0=0.57284,p2a=0.13100,p2b=0.05512 | 10.547252** |  |
|  |  | M1a | -11637.24401 | ω0=0.08048,ω1=1,ω2=85.86022,p0=0.65337,p2a=0.05222,p2b=0.02179 |  |  |
| Branch *d* | Bayesian | model A | -11636.22114 | ω0=0.08236,ω1=1,ω2=1,p0=0.41275,p2a=0.29980,p2b=0.07517 | 13.492258** | ***161*** |
|  |  | M1a | -11629.47501 | ω0=0.08229,ω1=1,ω2=∞,p0=0.68716,p2a=0.02945,p2b=0.01165 |  |  |
|  | ML | model A | -11640.90378 | ω0=0.08211,ω1=1,ω2=1,p0=0.42913,p2a=0.28417,p2b=0.11422 | 13.622996** |  |
|  |  | M1a | -11634.09228 | ω0=0.08200,ω1=1,ω2=∞,p0=0.68855,p2a=0.02815,p2b=0.01113 |  |  |
|  | MP | model A | -11639.61471 | ω0=0.08373,ω1=1,ω2=1,p0=0.00000,p2a=0.71676,p2b=0.28324 | 14.038464*** |  |
|  |  | M1a | -11632.59548 | ω0=0.08349,ω1=1,ω2=∞,p0=0.69235,p2a=0.02810,p2b=0.01090 |  |  |
|  | NJ | model A | -11646.31228 | ω0=0.08233,ω1=1,ω2=1,p0=0.42544,p2a=0.29177,p2b=0.11504 | 13.899016*** |  |
|  |  | M1a | -11639.36277 | ω0=0.08214,ω1=1,ω2=∞,p0=0.69205,p2a=0.02755,p2b=0.01073 |  |  |
| Branch *e* | Bayesian | model A | -11636.34815 | ω0=0.08240,ω1=1,ω2=1,p0=0.66378,p2a=0.04862,p2b=0.01963 | 28.072262*** | *8, 40,* ***205*** |
|  |  | M1a | -11622.31202 | ω0=0.08326,ω1=1,ω2=∞,p0=0.62333,p2a=0.08940,p2b=0.03603 |  |  |
|  | ML | model A | -11641.11839 | ω0=0.08223,ω1=1,ω2=1,p0=0.66645,p2a=0.04755,p2b=0.01905 | 27.543202*** |  |
|  |  | M1a | -11627.34679 | ω0=0.08331,ω1=1,ω2=∞,p0=0.62537,p2a=0.08826,p2b=0.03542 |  |  |
|  | MP | model A | -11639.40396 | ω0=0.08372,ω1=1,ω2=1,p0=0.66940,p2a=0.04909,p2b=0.01923 | 28.78421*** |  |
|  |  | M1a | -11625.01185 | ω0=0.08435,ω1=1,ω2=∞,p0=0.55809,p2a=0.08983,p2b=0.03534 |  |  |
|  | NJ | model A | -11646.11652 | ω0=0.08228,ω1=1,ω2=1,p0=0.66841,p2a=0.05066,p2b=0.01979 | 27.650384*** |  |
|  |  | M1a | -11632.29133 | ω0=0.08298,ω1=1,ω2=∞,p0=0.62950,p2a=0.08823,p2b=0.03470 |  |  |
| Branch *f* | Bayesian | model A | -11626.16627 | ω0=0.07957,ω1=1,ω2=1,p0=0.52960,p2a=0.16928,p2b=0.07294 | 24.188044*** | ***5****,* ***18****, 23,* ***26****,****36****,* ***38****,* ***43****, 49, 91,* ***114****, 140, 145,* ***160****,* ***166****, 190, 224,* ***238****,* ***257*** |
|  |  | M1a | -11614.07225 | ω0=0.08096,ω1=1,ω2=∞,p0=0.57731,p2a=0.11894,p2b=0.05189 |  |  |
|  | ML | model A | -11630.60816 | ω0=0.07923,ω1=1,ω2=1,p0=0.52522,p2a=0.17412,p2b=0.07486 | 23.29543*** |  |
|  |  | M1a | -11618.96044 | ω0=0.08050,ω1=1,ω2=∞,p0=0.57530,p2a=0.12099,p2b=0.05278 |  |  |
|  | MP | model A | -11631.6817 | ω0=0.08133,ω1=1,ω2=1,p0=0.53688,p2a=0.16728,p2b=0.07028 | 21.124286*** |  |
|  |  | M1a | -11621.11956 | ω0=0.08274,ω1=1,ω2=∞,p0=0.58785,p2a=0.11581,p2b=0.04877 |  |  |
|  | NJ | model A | -11638.71688 | ω0=0.08020,ω1=1,ω2=1,p0=0.52502,p2a=0.18036,p2b=0.07533 | 18.991448*** |  |
|  |  | M1a | -11629.22116 | ω0=0.08153,ω1=1,ω2=∞,p0=0.60077,p2a=0.10594,p2b=0.04397 |  |  |

^a^, After Bonferroni correction for multiple testing, branches *b*, *e* and *f* are still significant of all four tree topologies.

^b^, In*L* is the log-likelihood scores.

^c^, LRT to detect adaptive evolution. *** *P*<0.001; **0.001<*P*<0.01; * 0.01<*P*<0.05.

^d^, Those codons with posterior probabilities >99% are shown in boldface.
