# Supplementary material for: New insights into the evolution of subtilisin-like serine protease genes in Pezizomycotina
Source: BMC Evol Biol. 2010 Mar 9;10:68. doi: 10.1186/1471-2148-10-68 (PMC2848655; doi:10.1186/1471-2148-10-68)
Supplement: Additional file 5 — Results of one-way analysis of variance (ANOVA) and LSD-test using SPSS 14.0. The data of each treatment were carried out using one-way analysis of variance (ANOVA) in SPSS 14.0. Moreover, the LSD-test was also used for pairwise comparisons among the three treatments. A P value of less than 0.05 was considered as statistically significant. The results showed that the average amount of protein released into the supernatant in the protease treatment group (treatment a) was significantly higher than those with no protease (treatment b) or denatured protease (treatment c). Ia, The subtilisin-like serine protease PSP-3 produced by P. lilacinus on the eggs of the root-knot nematode Meloidogyne sp. IIb, The subtilisin-like serine protease PSP-3 produced by P. lilacinus on the eggs of the potato tuber moth P. opercullella. [file 1471-2148-10-68-S5.DOCX]

**Additional file 5-Results of one-way analysis of variance (ANOVA) and LSD-test using SPSS 14.0.**

**ANOVA**

|  |  | Sum of Squares | df | Mean Square | F | Sig. |
| --- | --- | --- | --- | --- | --- | --- |
| I^a^ | Between Groups | 0.084 | 2 | 0.042 | 97.907 | 0.000 |
|  | Within Groups | 0.022 | 51 | 0.000 |  |  |
|  | Total | 0.106 | 53 |  |  |  |
| II^b^ | Between Groups  Within Groups  Total | 0.074  0.025  0.099 | 2  51  53 | 0.037  0.000 | 74.539 | 0.000 |
|  |  |  |  |  |  |  |

LSD-test

|  | (I) group | (J) group | Mean Difference (I-J) | Std. Error | Sig. | 95% Confidence Interval | |
| --- | --- | --- | --- | --- | --- | --- | --- |
|  |  |  |  |  |  | Lower Bound | Upper Bound |
| I^a^ | a1 | b1 | 0.07416 | 0.00691 | 0.000 | 0.0603 | 0.0880 |
|  |  | c1 | 0.09089 | 0.00691 | 0.000 | 0.0770 | 0.1048 |
|  | b1 | a1 | -0.07416 | 0.00691 | 0.000 | -0.0880 | -0.0603 |
|  |  | c1 | 0.01673 | 0.00691 | 0.019 | 0.0028 | 0.0306 |
|  | c1 | a1 | -0.09089 | 0.00691 | 0.000 | -0.1048 | -0.0770 |
|  |  | b1 | -0.01673 | 0.00691 | 0.019 | -0.0306 | -0.0028 |
| II^b^ | a2 | b2 | 0.08132 | 0.00743 | 0.000 | 0.0664 | 0.0962 |
|  |  | c2 | 0.07541 | 0.00743 | 0.000 | 0.0605 | 0.0903 |
|  | b2 | a2 | -0.08132 | 0.00743 | 0.000 | -0.0962 | -0.0664 |
|  |  | c2 | -0.00591 | 0.00743 | 0.430 | -0.0208 | 0.0090 |
|  | c2 | a2 | -0.07541 | 0.00743 | 0.000 | -0.0903 | -0.0605 |
|  |  | b2 | 0.00591 | 0.00743 | 0.430 | -0.0090 | 0.0208 |

I^a^ , The subtilisin-like serine protease PSP-3 produced by *P. lilacinus* on the eggs of the root-knot nematode *Meloidogyne* sp.

II^b^, The subtilisin-like serine protease PSP-3 produced by *P. lilacinus* on the eggs of the potato tuber moth *P. opercullella.*
