# Supplementary material for: New insights into the evolution of subtilisin-like serine protease genes in Pezizomycotina
Source: BMC Evol Biol. 2010 Mar 9;10:68. doi: 10.1186/1471-2148-10-68 (PMC2848655; doi:10.1186/1471-2148-10-68)
Supplement: Additional file 7 — Detection of normal distribution with the Kolmogorov-Smirnov test and Shapiro-Wilk test in SPSS 14.0. The 18 data points of each treatment were checked for normal distribution with Kolmogorov-Smirnov test and Shapiro-Wilk test [64-66] in SPSS 14.0. The results showed that the data from the three treatments all approximated a normal distribution (P ≥ 0.05). Ia, The subtilisin-like serine protease PSP-3 produced by P. lilacinus on the eggs of the root-knot nematode Meloidogyne sp. IIb, The subtilisin-like serine protease PSP-3 produced by P. lilacinus on the eggs of the potato tuber moth P. opercullella. [file 1471-2148-10-68-S7.DOCX]

**Additional file 7-Detection of normal distribution with the Kolmogorov-Smirnov test and Shapiro-Wilk test in SPSS 14.0.**

|  |  | Kolmogorov-Smirnov test | | | Shapiro-Wilk test | | |
| --- | --- | --- | --- | --- | --- | --- | --- |
|  |  | Statistic | df | Sig. | Statistic | df | Sig. |
| I^a^ | treatment a1 | 0.119 | 18 | 0.200 | 0.980 | 18 | 0.953 |
|  | treatment b1 | 0.146 | 18 | 0.200 | 0.903 | 18 | 0.065 |
|  | treatment c1 | 0.190 | 18 | 0.086 | 0.945 | 18 | 0.354 |
| II^b^ | treatment a2 | 0.127 | 18 | 0.200 | 0.927 | 18 | 0.174 |
|  | treatment b2 | 0.162 | 18 | 0.200 | 0.918 | 18 | 0.121 |
|  | treatment c2 | 0.201 | 18 | 0.054 | 0.897 | 18 | 0.050 |

^a^, The subtilisin-like serine protease PSP-3 produced by *P. lilacinus* on the eggs of the root-knot nematode *Meloidogyne* sp.

^b^, The subtilisin-like serine protease PSP-3 produced by *P. lilacinus* on the eggs of the potato tuber moth *P. opercullella.*
